# Brain Mechanisms of Hypnosis

**DOI:** 10.3390/brainsci15020142

**Published:** 2025-01-31

**Authors:** Giuseppe De Benedittis

**Affiliations:** Department of Neurosurgery, University of Milano, 20122 Milano, Italy; giuseppe.debenedittis@unimi.it

Hypnosis is the longest-lasting of all psychotherapies and one of the oldest practiced methods for the control of pain and other stress-related chronic disorders.

Over the past few decades, hypnosis has matured into both a captivating subject for scientific inquiry and a potent clinical tool. This enviable history denotes and reflects its unsurpassed adaptive and evolutionary power [1].

Defined as ‘a state of consciousness involving focused attention and reduced awareness, characterized by an enhanced capacity for response to suggestion’ [2], hypnosis has gained significant traction in the fields of healthcare and education [3,4,5]. However, the neurophysiological and neuropsychological underpinnings of hypnotic experiences and responses remain elusive, leaving many fundamental questions about the essence of hypnosis and hypnotic responses unanswered. This Special Issue of *Brain Sci.* represents an outstanding effort to delve into and illuminate the neural mechanisms underlying hypnotic processes and responses, thereby advancing our comprehension of hypnotic practices.

To address these crucial questions and deepen our understanding of the brain mechanisms of hypnosis, we invited leading researchers and clinicians to share their insights, research findings, and recommendations for future work. This Special Issue presents eight peer-reviewed articles covering a wide range of topics, from the physiological correlates of hypnotizability to EEG correlates of hypnosis and hypnotic analgesia in both experimental and clinical settings (i.e., fibromyalgia patients). Other topics include cognitive neuroscience of hypnosis and hypnotizability, hypnotic modulation of the autonomic nervous system, and a calm contact technique rooted in endocrinological hypnotic mechanisms. The articles in this Special Issue provide critical analysis, cutting-edge research, clinical perspectives, and guidance for future research and clinical practice.

This Special Issue serves as a provisional overview of hypnosis’s past, present, and future. While primarily focused on experimental research, it also caters to clinicians. The Guest Editor aims to inspire both researchers and clinicians to delve deeper into the unexplored aspects of hypnosis, to pose novel questions, and to test uncharted ideas by bringing together these papers on current research. Rather than providing definitive answers, these papers stimulate critical thinking and encourage further exploration at the forefront of this fascinating and enigmatic field.

In taking stock of the papers in this Special Issue, Malloggi and Santarcangelo [6] (this volume) present a scoping review on the role of hypnotizability in the physiological variability of the general population, outlining that individuals with high, medium, and low hypnotizability scores display different cerebral functional differences (e.g., functional equivalence between imagery and perception/action, excitability of the motor cortex, interoceptive accuracy, and different control of blood supply) possibly related to the brain’s structural and functional characteristics. The authors postulate that these differences might influence prognostic and therapeutic outcomes in some medical disorders.

De Pascalis [7] (this volume) provides a comprehensive overview of the cognitive neuroscience of hypnosis and individual differences in hypnotizability, examining research utilizing functional magnetic resonance imaging (fMRI), positron emission tomography (PET), and electroencephalography (EEG). The reviewed findings on functional connectivity support the hypothesis that disruptions in the functional integration of the executive control network during hypnosis may be linked to altered subjective experiences of agency during the hypnotic response, suggesting a causal relationship between functional connectivity and cognitive, physiological, and behavioral responses. A promising avenue for future EEG research involves investigating how the integrated functioning of the frontal lobes with other cortical regions influences hypnosis and individual differences in hypnotizability during both waking and hypnotic states.

Varga and Nagy [8] (this volume) introduce the ‘calm contact’ technique, an imaginative scenario involving gentle contact with a loved one, emphasizing safety, calmness, and peaceful social connection. The theoretical underpinnings of the technique combine the brain mechanisms of stress reactions and hypnosis. Research has shown that hypnosis can reduce cortisol levels and elevate oxytocin levels. The beneficial effects of the ‘calm contact’ technique are linked to the concepts of social support and the psycho-affective effects of central oxytocin. Subjective reports from healthy volunteers illustrate the potential positive effects of the technique. The ‘calm contact’ technique may serve as an alternative or adjunct to the ‘safe place’ technique, leveraging recent findings on the neuroendocrinological mechanisms of hypnosis.

In addition to central top-down mechanisms, hypnosis exerts peripheral influence by modulating the autonomic nervous system (ANS). De Benedittis [9] (this volume) reviewed the relevant literature, analyzing studies employing common psychophysiological markers of ANS activity, such as heart rate variability (HRV), electrodermal activity (EDA), and the analgesia nociceptive index (ANI). Findings consistently demonstrate hypnosis’s ability to significantly impact ANS functions, reducing sympathetic activity and increasing parasympathetic tone. This effect is particularly pronounced during relaxation procedures and is influenced by factors such as hypnotizability and task conditions. This review highlights the potential of enhanced ANS modulation through hypnosis to optimize therapeutic outcomes in patients with psychosomatic disorders associated with ANS dysfunction.

Research indicates that therapeutic hypnosis is effective in reducing both acute and chronic pain. However, the underlying mechanisms of these effects remain largely unexplored. Jensen and Barrett’s review [10] (this volume) delves into the potential role of electroencephalogram (EEG)-assessed bandwidth power in identifying individuals who may benefit most from hypnotic analgesia and in understanding how these effects manifest. Their findings are discussed in light of the slow-wave hypothesis, which suggests that brain activity in slower-frequency bands (such as theta and alpha) may facilitate hypnotic responsiveness. The results of their research align generally with this hypothesis.

Hypnosis offers significant potential for managing fibromyalgia and chronic pain. While its feasibility and efficacy are well established, the underlying mechanisms remain elusive. Behavioral research suggests that an altered sense of agency may contribute to pain reduction during hypnosis. Building on these findings, this innovative study by Kumar Govindaiah et al. [11] (this volume) aims to investigate neural activity during hypnosis in fibromyalgia patients using high-density electroencephalography (EEG) and self-reported measures. Neural oscillations revealed increased theta power in left parietal and occipital electrodes, increased beta power in frontal and left temporal electrodes, and increased slow-gamma power in frontal and left-parietal electrodes during hypnosis. Functional connectivity analysis using coherence measures indicated decreased connectivity between frontal electrodes. Key findings demonstrate substantial modifications in neural oscillations and brain functional connectivity, indicating potential electrophysiological biomarkers of hypnotic state in this specific patient population.

Hypnotic phenomena exhibit significant inter-individual variability, with some individuals demonstrating strong responses to hypnotic suggestions, while others show limited susceptibility. Recent neurophysiological studies have linked this variability to distinct neural characteristics. However, our understanding of the time-varying nature of these neural features and their relationship to hypnotic susceptibility remains limited. Landry et al. [12] (this volume) conducted a time-resolved analysis of rhythmic alpha peaks and arrhythmic components of the EEG spectrum both before and after hypnotic induction. Using multivariate pattern classification, they investigated whether these non-stationary neural features could distinguish between individuals with high and low susceptibility to hypnosis. The results show that variations in the alpha center frequency are indicative of hypnotic susceptibility, but this discrimination is only evident during hypnosis. Highly hypnotizable individuals exhibit higher variability of alpha peak center frequency. These findings highlight the dynamic changes in neural states related to alpha peak frequency as a central neurophysiological feature of hypnosis and hypnotic susceptibility.

Managing anxiety and behavior during pediatric dental procedures remains a significant challenge. Rienhoff et al. [13] (this volume) investigated the combined effects of ibuprofen with midazolam sedation, alongside behavioral management and clinical hypnosis, to improve patient cooperation and reduce post-treatment pain. A retrospective cohort study of 311 children was conducted. Patients received either midazolam with ibuprofen (*n* = 156) or midazolam only (*n* = 155). Ibuprofen did not significantly improve behavior during procedures, suggesting that pharmacological pain management alone is insufficient to address behavioral challenges. However, ibuprofen significantly reduced post-treatment pain, with 7.2% of cases reporting pain in the non-ibuprofen group compared to none in the ibuprofen group (*p* < 0.05). This study underscores the importance of integrating sedation with behavioral strategies, such as clinical hypnosis, to manage anxiety, improve patient cooperation, and enhance overall treatment outcomes in pediatric dentistry. Further research is needed to optimize these strategies and validate them in a prospective setting.

This Special Issue offers a comprehensive review of the challenges and opportunities facing the field of hypnosis, featuring contributions from leading theorists and researchers. Their articles delve into definitions, theory, research, and practice, addressing questions such as the following: What do we know? What can we do? What remains unknown? And what are we still learning or striving to achieve?

The Guest Editor extends sincere gratitude to all of the authors for their valuable contributions and to the excellent reviewers for their insightful feedback, which was instrumental in bringing this Special Issue to fruition. Their research and insights can deepen our understanding of the multifaceted nature of hypnosis, stimulate innovative research directions, and explore the potential for integrating hypnosis into various interventions.

The future of hypnosis research hinges on the ability of researchers to bridge the gap between hypnosis and broader fields of knowledge. By capitalizing on hypnosis’s potential to extend major theories in sociocognitive psychology, the neural basis of consciousness, and its applications in medicine, psychology, and psychiatry, we can advance our understanding and utilization of this powerful tool.
